# An In Vitro Analysis of the Effects of Intravenous Lipid Emulsion on Free and Total Local Anaesthetic Concentrations in Human Blood and Plasma

**DOI:** 10.1155/2014/236520

**Published:** 2014-11-05

**Authors:** Louise Ann Clark, Jochen Beyer, Andis Graudins

**Affiliations:** ^1^Monash Health Emergency Medicine Program, Monash Medical Centre, Clayton Road, Clayton, VIC 3168, Australia; ^2^Department of Forensic Toxicology, Institute of Forensic Medicine, Kantonsspital St. Gallen, Rorschacher Straße 95, Building 11, 9007 St. Gallen, Switzerland; ^3^Department of Forensic Medicine, Victorian Institute of Forensic Medicine, Monash University, 57-83 Kavanagh Street, Southbank, VIC 3006, Australia; ^4^School of Clinical Sciences at Monash Health, Monash University, Clayton, VIC 3168, Australia

## Abstract

*Background*. Intravenous lipid emulsion (ILE) is recommended as a “rescue” treatment for local anaesthetic (LA) toxicity. A purported mechanism of action suggests that lipophilic LAs are sequestered into an intravascular “lipid-sink,” thus reducing free drug concentration. There is limited data available correlating the effects of ILE on LAs. *Aims*. To compare the in vitro effect of ILE on LA concentrations in human blood/plasma and to correlate this reduction to LA lipophilicity. *Method*. One of four LAs (bupivacaine-most lipophilic-4 mg/L, ropivacaine-6 mg/L, lignocaine-14 mg/L, and prilocaine-least lipophilic-7 mg/L) was spiked into plasma or whole blood. ILE or control-buffer was added. Plasma was centrifuged to separate ILE and total-LA concentration assayed from the lipid-free fraction. Whole blood underwent equilibrium dialysis and free-LA concentration was measured. Percent reduction in LA concentration from control was compared between the LAs and correlated with lipophilicity. *Results*. ILE caused a significant reduction in total and free bupivacaine concentration compared with the other LAs. Ropivacaine had the least reduction in concentration, despite a lipophilicity similar to bupivacaine. The reduction in LA concentration correlated to increasing lipophilicity when ropivacaine was excluded from analysis. *Conclusion*. In this first in vitro model assessing both free- and total-LA concentrations exposed to ILE in human blood/plasma, ILE effect was linearly correlated with increasing lipophilicity for all but ropivacaine.

## 1. Introduction

Local anaesthetics (LAs) are commonly used in emergency medicine and anaesthetic practice. Inadvertent systemic administration of these agents may lead to local anaesthetic systemic toxicity (LAST), manifest by malignant cardiac arrhythmias, cardiac arrest, and/or neurotoxicity. These may be refractory to conventional resuscitation treatments.

Intravenous lipid emulsion (ILE), a heterogeneous mixture of fat molecules in an emulsion, is usually utilized as a parenteral nutritional agent. Based on previous in vitro and in vivo studies suggesting a positive effect on bupivacaine cardiotoxicity, ILE is currently recommended as an antidote for LAST from all LA agents. Currently, various national anaesthetic colleges endorse ILE as an adjunctive treatment to standard resuscitative therapy following suspected LAST [1].

The mechanism of action of ILE in LAST is controversial. The most widely accepted mechanism proposes that it provides a “lipid sink” or compartment within plasma into which lipophilic drugs are sequestered [2]. It is theorized that this results in a reduction in plasma-free drug concentration resulting in a concentration gradient away from target tissues where toxicity is manifest. It is likely that the degree of binding to ILE may vary with each LA, given the varying lipid solubility of different LAs. A knowledge gap exists as to the effectiveness of ILE in binding LA agents in both in vitro and in vivo models of drug toxicity as well as in the clinical setting.

## 2. Materials, Methods, and Methodology

This is an unblinded in vitro laboratory-based assessment of the effects of ILE on (1) total-LA drug concentration in human plasma and (2) free-LA drug concentration in whole human blood.

### 2.1. Materials

Human whole blood and plasma were donated by the Australian Red Cross Blood Bank, Southbank, Melbourne, Victoria.

Local anaesthetic agents used were therapeutic formulations of bupivacaine 0.5% (Bupivacaine, Pfizer, West Ryde, Australia), lignocaine 1% (Xylocaine, AstraZeneca, North Ryde, Australia), prilocaine 0.5% (Citanest, AstraZeneca, North Ryde, Australia), and ropivacaine 0.5% (Naropin, AstraZeneca, North Ryde, Australia). Drug concentrations were determined as causing LA toxicity based upon literature reported concentrations that have been associated with significant cardiovascular or central nervous system toxicity or mortality [3]. These are summarised in Table 1 with the associated pharmacokinetic parameters for the particular LAs.

ILE was a commercially available product, provided as a 20% formulation of Intralipid 20% (Fresenius Kabi Australia Pty Limited).

Rapid equilibrium devices and the associated base plate required for equilibrium dialysis experiments used to determine free drug concentration in whole blood were sourced from Thermo Scientific, Pierce Research, Rockford, IL, USA.

### 2.2. Assessment of Total Drug Concentrations of LAs in Human Plasma

One of bupivacaine, prilocaine, ropivacaine, or lignocaine was added to human plasma. For each LA agent, experiments were performed in triplicate. Human plasma rather than whole blood was utilized as we had previously determined that red cell haemolysis would result by centrifugation when attempting to separate the lipid phase from the blood phase.

The effect of ILE on LA concentration was assessed firstly for all agents at a concentration approximating 4 mg/L. A second set of assays were performed using a higher LA concentration for prilocaine, ropivacaine, and lignocaine to approximate serum concentration reported in cases of severe LAST associated with cardiac arrest. Drug concentrations used in the experiments are summarized in Table 1.

The pH of human plasma, blood, and Intralipid was determined using a clinical blood gas analysis machine as 7.2, 7.0, and 6.0, respectively.

Intralipid 20% or phosphate buffered saline (PBS) was added to plasma samples containing LA to make a final total volume of 1 mL at three concentrations—2% (20 *μ*L/mL), 10% (100 *μ*L/mL), and 20% (200 *μ*L/mL).

Samples were allowed to equilibrate on a temperature controlled orbital shaker (Lomb Scientific, Australia) for 30 minutes at 60 rpm and 37°C. The samples were then centrifuged for 1 hour at 13,000 rpm and temperature of 37°C (Eppendorf Centrifuge 5115 R, Thermo Fisher Scientific, Australia). The plasma portion of samples was carefully aspirated from the lipid layer supernatant and then frozen and stored at −20°C for later batch analysis at the Victorian Institute of Forensic Medicine (VIFM).

### 2.3. Assessment of Free Drug Concentrations in Whole Human Blood with Varying Concentrations of ILE by Equilibrium Dialysis

Only the higher LA concentrations were used for the whole blood assays (Table 1). ILE or PBS was added to a final volume of 1 mL in the same concentrations as in the plasma experiments. The pH of stored whole human blood was determined to be 7.0 by blood gas machine estimation. The samples were incubated for 30 minutes at 37°C on an orbital shaker, before introduction into a rapid equilibrium dialysis (RED) chamber.

Equilibrium dialysis was undertaken using the RED devices. These have been validated previously for analysis of free drug concentration in whole blood [4]. The device consists of two side-by-side chambers separated by a dialysis membrane. One chamber contains the sample and the adjacent chamber contains a sample buffer. The RED devices are placed in a chamber on the base plate where multiple assays can be performed simultaneously.

PBS was utilised in these experiments as the dialysate in the buffer chamber. Blood containing LA was incubated with either ILE or PBS, and an equivalent volume of PBS was introduced into the adjacent chamber.

The samples were incubated for 6 hours (at 37°C) to allow for equilibration of free drug not bound to plasma proteins in blood with the PBS in the buffer chamber. The concentration of drug measured in the buffer chamber reflects the free drug concentrations in the blood chamber. Following incubation, the contents of each chamber were aspirated and frozen at −20°C for later batch analysis.

### 2.4. Drug Concentration Assay

At the Victorian Institute of Forensic Medicine (VIFM), samples were prepared for drug concentration analysis by standard extraction methods. After thawing, these were mixed on a mini Vortex for 5 seconds. Following mixing, 180 *μ*L of each sample was transferred into new sample tubes. Similarly, 160 *μ*L of each of the calibrators (plasma or whole blood samples, made up to a final volume of 1 mL with either blood, ILE, or PBS; spiked at VIFM with a standardized known concentration of drug) was transferred into new sample tubes. As internal standard, 20 *μ*L of trimipramine was added at a concentration of 1 mg/mL, to all samples and calibrators.

A stock solution of each local anaesthetic was made up to a known concentration (bupivacaine 4 mg/L, lignocaine 4 and 14 mg/L, prilocaine 4 and 7 mg/L, and ropivacaine 4 and 6 mg/L) and 20 *μ*L was added to each of the calibrator samples. All samples at this point had a total volume of 200 *μ*L within each Eppendorf tube.

Trizma buffer (0.2 mL) and 1-chlorobutane (1 mL) were added to the samples and these were mixed thoroughly. These solvents extract drug from the samples by denaturing plasma proteins and lysing red blood cells to release protein bound drug. The samples were extracted for 10 minutes on a VXR basic Vibrax shaker (Sigma Aldrich, Sydney, Australia) at 2500 rpm and then centrifuged for 5 minutes at 13,000 rpm. The clear solvent layer containing drug was transferred into clean glass autosampler vials. The vials were evaporated to dryness on a Ratek dry down sample concentrator (Thermo Fisher Scientific, Boronia, Australia) for 20 minutes. Samples were then reconstituted in 100 *μ*L of methanol.

Drug concentrations were determined utilizing gas chromatography mass spectrometry (GC-MS), a technique validated for the reliable detection of the local anaesthetic agents being assayed [5]. The prepared samples were analysed using a GC-MS QP2010 series (Shimadzu, Australia). The GC-MS generated a mass spectra peak for each sample. The concentrations were calculated using the peak area ratios of each drug and their respective extracted calibration curves.

### 2.5. Data Collection Analysis and Statistics

All experiments were performed in triplicate and results entered into an Excel spreadsheet. Graphical representation of results was undertaken using GraphPad Prism, 5.0 (GraphPad Software, Ltd).

Percent reductions in LA concentration for each drug are compared at each ILE concentration for both total and free drug concentration experiments. These are reported graphically as mean ± SEM. Differences in percent reduction of LA concentration were compared at each ILE concentration using a 1-way ANOVA with a Bonferroni posttest analysis.

Finally, correlation between percent reduction in total and free-LA concentrations with ILE treatment and respective lipophilic index (Log *P*_(octanol)_) was undertaken using linear regression analysis in GraphPad Prism using the higher LA concentration data.

## 3. Results

### 3.1. Effects of ILE on Total Drug Concentrations in Human Plasma

**Figure 1:**
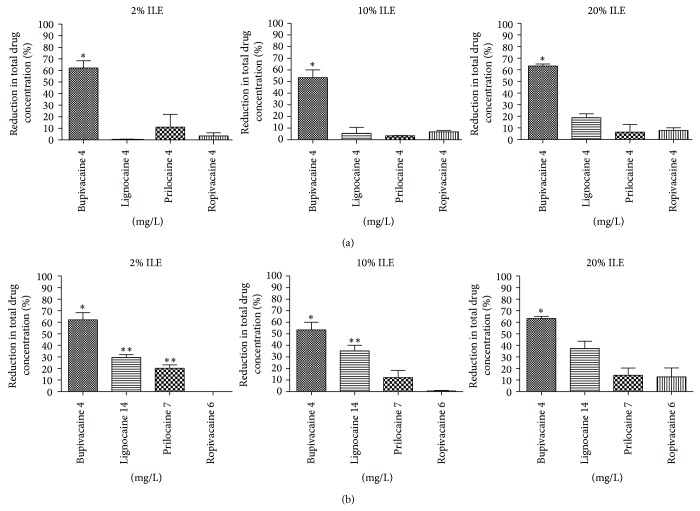
Comparison of percent reduction in total-LA concentrations in human plasma treated with ILE at three concentrations (2%, 10%, and 20%). (a) Local anaesthetic concentration similar for all four agents (4 mg/L). Bupivacaine showed a significant reduction in concentration compared to all three other LAs. 2% ILE: *P* = .0004.  ^*^Bupivacaine significant compared to all other LAs. 10% ILE: *P* < .0001.  ^*^Bupivacaine significant compared to all other LAs. 20% ILE: *P* < .0001.  ^*^Bupivacaine significant compared to all other LAs. (b) Varying LA concentration for each agent dependent upon reported concentrations associated with clinical cases of severe CVS toxicity. Bupivacaine showed the greatest percent reduction in concentration across the three ILE groups. This was significant compared to all other LAs in all three ILE groups. 2% ILE: *P* < .0001 one-way ANOVA. Bonferroni's posttest:  ^*^bupivacaine significant compared to all other LAs,  ^**^lignocaine and prilocaine significant to ropivacaine. 10% ILE: *P* = .0007 one-way ANOVA. Bonferroni's posttest:  ^*^bupivacaine significant compared to all other LAs,  ^**^lignocaine significant to ropivacaine. 20% ILE: *P* = .0009 one-way ANOVA. Bonferroni's posttest:  ^*^bupivacaine significant compared to all other LAs. Mean ± S.E.M = mean values ± standard error of means of three experiments.

Total bupivacaine concentration fell the most with ILE in plasma at all three ILE concentrations, with the mean percent reduction approximately 60%. When the percent reduction was compared with the other LA agents at each lipid concentration a statistically significant reduction was seen with bupivacaine compared to all other LAs (Figure 1). Bupivacaine concentration was reduced to a similar degree at all three ILE concentrations. There was a greater reduction in LA concentration with the other agents at the higher plasma concentration. Notably, ropivacaine had the smallest reduction in plasma concentrations of all four drugs at the higher concentration.

### 3.2. Effects of ILE on Free Drug Concentration in Human Whole Blood Using Equilibrium Dialysis

**Figure 2:**
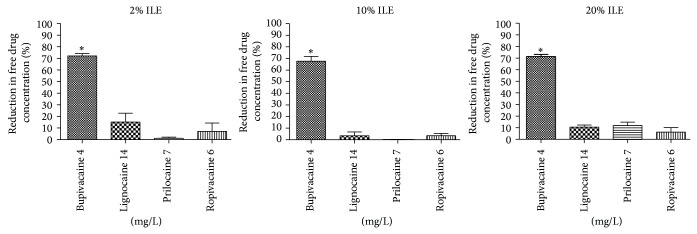
Percent reduction in free-LA concentrations after equilibrium dialysis of whole human blood treated with ILE at three concentrations (2%, 10%, and 20%). Only the higher LA concentration was used for each agent for assessment of the effect of ILE on free drug. Bupivacaine showed the most consistent percent reduction in its concentration (50–60%) across the three ILE groups. Bupivacaine showed a significant reduction in concentration compared to all other LA agents in all three ILE groups. 2% ILE: *P* < .0001 one-way ANOVA. Bonferroni's posttest:  ^*^bupivacaine significant compared to all other LAs. 10% ILE: *P* < .0001 one-way ANOVA. Bonferroni's posttest:  ^*^bupivacaine significant compared to all other LAs. 20% ILE: *P* < .0001 one-way ANOVA. Bonferroni's posttest:  ^*^bupivacaine significant compared to all other LAs. Mean ± S.E.M = mean values ± standard error of means of three experiments.

Bupivacaine showed a statistically significant reduction in percent fall of drug concentration compared to the other LA agents at all ILE concentrations (Figure 2). As seen with the plasma experiments, the reduction in drug concentration for all the LA agents was consistent across all ILE treatment groups and did not increase with increasing ILE concentration.

Notably, there was minimal reduction in free ropivacaine concentration in any ILE group. This parallels the results observed with total ropivacaine concentration seen in the plasma experiments at the higher toxic concentration used.

### 3.3. Changes in LA Concentration and the Relationship with the Lipophilic Index

We graphically compared the percent reduction in LA concentration at the higher drug concentrations to each drug's lipophilic index (Log *P*_(octanol)_).

**Figure 3:**
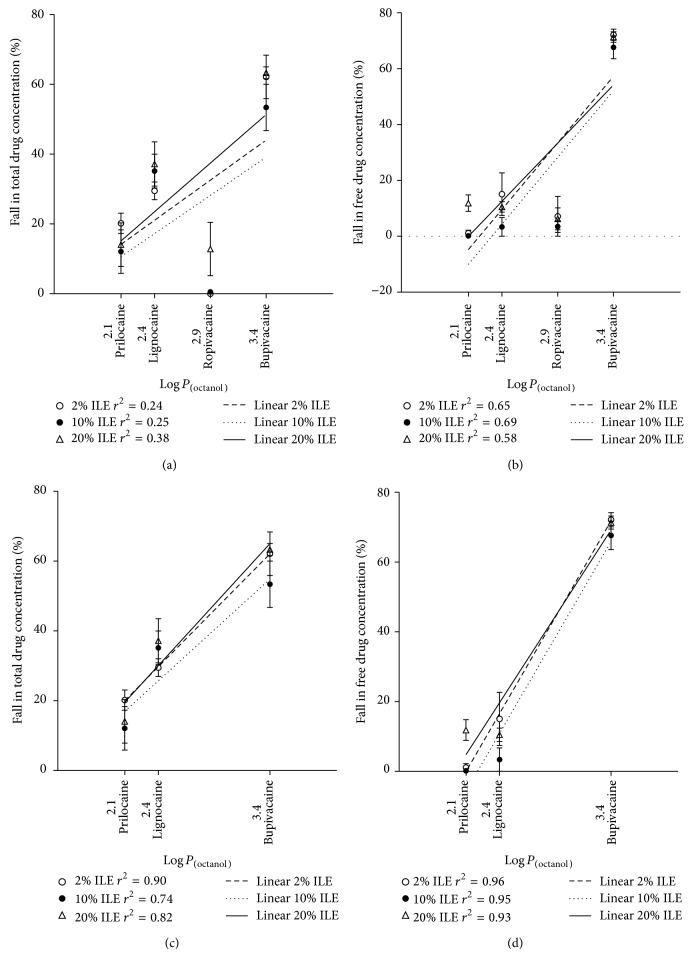
Comparison of the reduction in (a) total drug concentration of LAs in human plasma and (b) free drug concentration of LAs in whole human blood with the lipophilic index of each drug (Log *P*_(octanol)_) with linear regression lines included when all four local anaesthetics are displayed. With the removal of ropivacaine data, a linear correlation using linear regression analysis was observed between reduction of local anaesthetic concentration and Log *P*_(octanol)_ for total drug concentration (c) and free drug concentration (d). There was no linear relationship between increasing Log *P*_(octanol)_ and greater binding to lipid emulsion by the local anaesthetics. Bupivacaine showed the greatest reduction in total and free drug concentrations and has the highest lipophilic index. Ropivacaine showed unexpectedly low reductions of drug concentrations with ILE treatment despite having the second highest lipophilic index.

When all four drugs were included, there was no correlation between drug concentration and increasing lipophilic index for total drug in plasma or free drug in blood by dialysis (Figures 3(a) and 3(b)). Bupivacaine has the greatest Log *P*_(octanol)_ and had the greatest effect on total and free drug concentration. Ropivacaine did not follow the linear trend that would be expected by its relatively high lipophilicity.

Reanalysis of the comparison between lipophilic indices and the percent reduction in drug concentration was undertaken with the exclusion of ropivacaine. A linear correlation was observed between increasing Log *P*_(octanol)_ and reduction in drug concentration for the remaining three LAs using linear regression analysis (Figures 3(c) and 3(d)).

## 4. Discussion

We describe the effect of varying concentrations of ILE on total and free-LA drug concentration in human plasma and whole blood, respectively. Of note, this is the first report that examines total drug concentration and free drug concentration for LAs in a model utilizing human blood products.

Bupivacaine showed the greatest percent reduction in both total and free drug concentrations in comparison with the other LAs. This was seen with ILE at all concentrations assessed and correlates with the fact that this is the most lipophilic of LA agents tested. A similar, albeit less significant, effect was seen with the other LAs. However, ropivacaine, surprisingly, displayed the least reduction in plasma drug concentration and very low reductions in free drug concentration despite having the second highest lipophilicity index of the LAs assayed.

When comparing the reduction in LA concentration with ILE treatment and the lipophilicity index of each agent, we initially did not observe a linear correlation between these two parameters. However, if the data for ropivacaine is excluded from this analysis, a linear correlation is evident for the other LAs.

The first concentration of ILE was chosen as it approximates the blood concentration that is likely to be achieved in vivo after a loading dose of 1.5 mL/kg of Intralipid. This equates to approximately 3.5% of the total plasma volume of the body. The 10% concentration was used to approximate the concentration of ILE in the blood that might be seen after a loading dose and a subsequent infusion of a further 300–400 mL of Intralipid as recommended in guidelines such as those found at http://www.lipidrescue.org. The 20% concentration is likely to represent one that is only achievable when more than a litre of ILE is administered intravenously in the clinical setting.

Other in vitro studies utilizing non-LA lipophilic drugs have illustrated a positive correlation between lipophilicity and the effects of ILE on drug concentration [6, 7]. However, in our study, the results for ropivacaine were inconsistent with the association between increasing lipophilicity and ILE binding. Ropivacaine has the second highest lipophilic index of the LAs tested. If the primary mechanism of ILE effect in mitigating LA toxicity is the sequestration of drug into the lipid phase, it would be expected that ropivacaine would bind ILE to a greater degree than observed. Previous studies examining the effect of ILE on ropivacaine concentration in buffer solutions have also observed that the decrease in ropivacaine concentration is less than that seen with bupivacaine despite similar lipophilic indices [6, 8, 9]. Mazoit et al. [8] observed a 60% reduction in bupivacaine concentration in buffer solution treated with 1% ILE as opposed to a 30% reduction in ropivacaine concentration in the same buffer model. Similarly, Evans et al. [9] demonstrated a 40% reduction in bupivacaine concentration after addition of ILE, whereas ropivacaine binding to ILE was more variable and was reduced by approximately 20%. French et al. [6] examined the fall in concentration of three LAs (bupivacaine, ropivacaine, and mepivacaine) after mixing with ILE. This study found that the LA concentration of bupivacaine, ropivacaine, and mepivacaine decreased by 18%, 7%, and 12%, respectively, despite mepivacaine having a lower octanol/water coefficient (1.9) than ropivacaine (2.9). These observations parallel our results.

We did not maintain pH of plasma or blood in the normal physiological range. The pH of the stored blood and plasma we used was 7.0 and 7.2, respectively. As observed in other in vitro models [8], falling pH may be associated with reduced effectiveness of ILE binding to LAs. Mazoit et al. noted that the effect of lipid emulsion on reduction in ropivacaine and bupivacaine concentration was reduced similarly with a decrease in pH from 7.4 to 7.0 [8]. Hence, it is possible that binding to ILE at lower pH may have affected the four LAs similarly thus not altering the relative effect of ILE across the LAs tested in this report. In the clinical scenario of LAST with cardiac arrest or severe hypotension, patients frequently have associated acidaemia. As such, we felt it would be important to explore lipid binding to LAs within an acidaemic environment, to more closely replicate the likely clinical state. Given that our system was maintained at a lower than physiological pH, this may explain the poor result with ropivacaine binding to ILE. However, it may also indicate that ILE may not work as well in the setting of an acidaemic patient.

Bupivacaine and ropivacaine have a similar pharmacokinetic profile, with the exception that ropivacaine has a larger volume of distribution (Table 1). This pharmacokinetic property will not influence results in an in vitro model and is unlikely to explain the results seen with ropivacaine.

Alternate theories for the mechanism of action of ILE in bupivacaine toxicity have been suggested. Firstly, bupivacaine inhibits mitochondrial acylcarnitine transferase activity and subsequent migration of free fatty acids (FFAs) into these organelles. Provision of higher extracellular concentrations of FFAs by ILE administration is theorized to increase the delivery of FFAs to the myocardium and cellular function [10]. Secondly, FFAs found in ILE have been shown to inhibit binding of bupivacaine to myocardial cell voltage-gated Na^+^-channels, reducing use-dependent block and inhibition of ion fluxes by this LA [11]. Both mechanisms may explain the positive effects of ILE in bupivacaine toxicity. However, none of these mechanisms have been investigated in LA toxicity resulting from other agents, including ropivacaine, and it is uncertain whether similar mechanisms are exerted on the myocardium by ILE for these agents. Case reports of ILE use in LAST from lignocaine, prilocaine, ropivacaine, and other LAs exist. The indications for, timing of, and response to ILE therapy are not well documented in most case reports [12, 13].

## 5. Conclusion

In this in vitro model, bupivacaine, the most lipophilic LA, exhibited the greatest reduction in total and free drug concentration when treated with ILE compared to the other LAs. Despite a comparable lipophilicity, ropivacaine concentration did not decrease to a similar degree when exposed to lipid emulsion. This study suggests that there was a correlation between reduction in LA concentration and increasing drug lipophilicity; however it does not prove causality as the mechanism for ILE effect in LA poisoning.

## Figures and Tables

**Table 1:** Summary of local anaesthetic concentrations utilised in this study and pharmacokinetic properties of the various agents used.

Local anaesthetic	Concentrations utilised (mg/L)	log⁡*P*_(octanol/water)_ (lipid partition coefficient)	p*K_a_*	Protein binding (%)	Volume of distribution (L/kg)
Prilocaine	4, 7	2.1	7	50	0.7–4.4
Lignocaine	4, 14	2.4	7.9	70	1-2
Ropivacaine	4, 6	2.9	8.16	94	41–59
Bupivacaine	4	3.4	8.1	90	1

## Abbreviations

GC-MS: Gas chromatography mass spectrometry
ILE: Intravenous lipid emulsion
LA: Local anaesthetic
$\text{Log}\;P_{\left(\text{octanol}\right)}$: Logarithmic expression of lipophilic partition coefficient of drugs in octanol and water
PBS: Phosphate buffered saline
RED: Rapid equilibrium dialysis
VIFM: Victorian Institute of Forensic Medicine.
